# Disruption of cell wall fatty acid biosynthesis in *Mycobacterium tuberculosis *using a graph theoretic approach

**DOI:** 10.1186/1742-4682-8-5

**Published:** 2011-03-31

**Authors:** Veeky Baths, Utpal Roy, Tarkeshwar Singh

**Affiliations:** 1 Department of Biological Sciences, Birla Institute of Technology & Science (BITS) Pilani K K BIRLA Goa Campus, Goa 403 726, India; 2 Department of Mathematics, Birla Institute of Technology & Science (BITS) Pilani K K BIRLA Goa Campus, Goa 403 726, India

## Abstract

Fatty acid biosynthesis of *Mycobacterium tuberculosis *was analyzed using graph theory and influential (impacting) proteins were identified. The graphs (digraphs) representing this biological network provide information concerning the connectivity of each protein or metabolite in a given pathway, providing an insight into the importance of various components in the pathway, and this can be quantitatively analyzed. Using a graph theoretic algorithm, the most influential set of proteins (sets of {1, 2, 3}, etc.), which when eliminated could cause a significant impact on the biosynthetic pathway, were identified. This set of proteins could serve as drug targets. In the present study, the metabolic network of *Mycobacterium tuberculosis *was constructed and the fatty acid biosynthesis pathway was analyzed for potential drug targeting. The metabolic network was constructed using the KEGG LIGAND database and subjected to graph theoretical analysis. The nearness index of a protein was used to determine the influence of the said protein on other components in the network, allowing the proteins in a pathway to be ordered according to their nearness indices. A method for identifying the most strategic nodes to target for disrupting the metabolic networks is proposed, aiding the development of new drugs to combat this deadly disease.

## Background

The complete genome sequence of the best-characterized strain of *Mycobacterium tuberculosis*, H37Rv, has been determined and analyzed, improving understanding of the biology of this slow-growing pathogen and aiding the development of new prophylactic and therapeutic interventions [1]. The genome information concerning the H37Rv strain was used in this study.

Graph representation of the entire metabolism of the bacterium demonstrates the various clusters of proteins and their connectivity [2]. Furthermore, analyzing a well-connected cluster of proteins linked to several pathways enables the specific pathway concerned with mycolic acid synthesis to be targeted [3]. The bacterium possesses a thick layer of lipid on the outer surface that protects it from noxious chemicals and the host's immune system [3]; these lipids are also present in the *Corynebacterium-Mycobacterium-Nocardia *group. They give rise to important characteristics including resistance to chemical injury and dehydration, low permeability to antibiotics, virulence, acid-fast staining and the ability to persist within a host. Mycolic acids are the major constituents of this protective layer [4] and they play important roles as structural components of the cell wall and envelope [5]. In particular, the cyclopropane rings of mycolic acids in *M. tuberculosis *contribute to the structural integrity of the cell wall complex and protect the bacillus from oxidative stress (hydrogen peroxide) [3]. The lack of drug compliance, the appearance of multi-drug-resistant strains and the AIDS epidemic are factors that have led to a resurgence of tuberculosis infection. Drug resistance follows inadequate compliance, and AIDS patients with a weakened immune system are very susceptible to *M. tuberculosis *and it is a common cause of death [3].

Various graph theory approaches have been used to analyze metabolism in bacteria. In the present study, construction of a metabolome-based reaction network of *Mycobacterium tuberculosis *was attempted using the KEGG LIGAND database, and graph spectral analysis of the network was carried out to identify hubs and the sub-clustering of reactions. Another approach used for drug targeting was the identification of the 'load points' and 'choke points' in metabolic networks (graphs representing metabolism). In order to identify potential drug targets (based on the biochemical lethality of metabolic networks), the concept of choke points and load points was used to identify enzymes (edges) that uniquely consume or produce a particular metabolite (node) [6]. Complete genome sequences describe the range of metabolic reactions possible for an organism, but they cannot quantitatively describe the behavior of these reactions. In this study, a novel method for modeling metabolic states using whole cell measurements of gene expression is presented. The method, called E-Flux (a combination of Flux and Expression), extends the technique of Flux Balance Analysis by modeling maximum flux constraints as a function of measured gene expression [7].

## Methods

### A Graph Theoretic Approach

An ordered pair G = (V, E), where V is a non-empty set whose elements are called vertices (nodes or points), and E is a set of two distinct elements that are a subset of V, whose elements are called edges (links or lines) [8]. Furthermore, a graph G is said to be finite if V is finite, otherwise it is termed infinite. Two vertices, u and v, are said to be adjacent in G if u and v are joined by an edge, otherwise they are non-adjacent. An edge e and a vertex u are said to be incident if u is one of the end vertices of the edge e. Two edges, e_1 _and e_2_, are said to be adjacent if both have the same end vertices. The cardinality of V is denoted by |V| = p: = number of vertices in V, is called the order of the graph G. Similarly, the cardinality of E i.e. |E| = q is called the size of G. The graph can be represented using diagrams and matrices. On the basis of adjacency and incidence relationships among edges and vertices, adjacency and incidence matrices can be determined [8].

The adjacency matrix of a graph G, with vertex set V = {v_1_,v_2_,...,v_p_}, is denoted A(G) = [a_i,j_]_p × p_, where a_i,j _= 1 if there is an edge between v_i _to v_j _and 0 otherwise. It is a binary (0, 1) - square symmetric matrix. The incidence matrix of a graph G is denoted I (G) = [b_i, j_] _p × q_, where b_i, j _= 1 if e_i _is incident to v_i_, and 0 otherwise. A graph H = (V_1_, E_1_) is said to be a sub-graph of the graph G = (V, E) if V_1 _is a subset of V and E_1 _is a subset of E. When V_1 _= V then the sub-graph H is termed a spanning sub-graph of G. A directed graph (digraph in short) D is an ordered pair D = (V, A), where V is known as a vertex set of D and A is an ordered pair of two distinct elements of V, known as an arc set of D. The order and size of a digraph D is the number of vertices and arcs in D, respectively. In a digraph, if there is an arc a = (u, v), then u is the initial vertex and v is the terminal vertex of the arc a. A graph G is said to be connected if one vertex can be reached from another vertex by a path (alternating sequence of vertices and edges, i.e. u = u_0_, e_0_, u_1_, e_1_, u_2_,..., u_n-1_, e_n-1_, u_n _= v), otherwise it is considered to be disconnected.

A digraph is said to be strongly connected if a vertex of D can be reached from other vertices of D by a directed path. A digraph D is said to be weakly connected if its underlying graph (undirected graph) is connected. Deletion of a vertex v from the digraph D (graph G) refers to the removal of a vertex v from V and all arcs (edges) for which v is either the initial vertex or the terminal vertex of an arc in D. Deletion of an arc (edge) from a digraph (graph) refers to the removal of the arc (edge) from the digraph (graph). If S is a subset of the vertex set V of the digraph (graph), then D - S is the digraph (graph) obtained by removing vertices of S and arcs (edges) for which one of the end vertices are in S from the D. If there are more than two connected components, then S is referred to as the separating set of D. Let [S, V-S] = {(u,v): u ε S and v ε V-S}; if all arcs (edges) of [S, V-S] are removed from the digraph (graph) and the resultant digraph (graph) is not connected, then [S, V-S] is termed an edge-cut of D.

**Figure 1:**
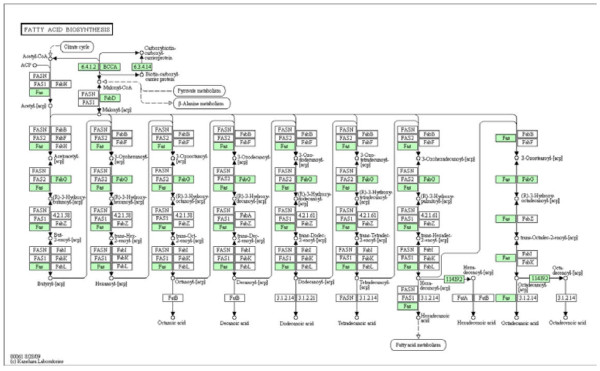
**Fatty acid Biosynthesis (Source KEGG pathway database)**. Fatty Acid Synthesis Pathway in *Mycobacterium tuberculosis *H37rv. The pathway was downloaded from the KEGG Database. Starting from Acetyl-CoA at the bottom left of the figure, the metabolites are numbered left to right and then bottom to top.

**Figure 2:**
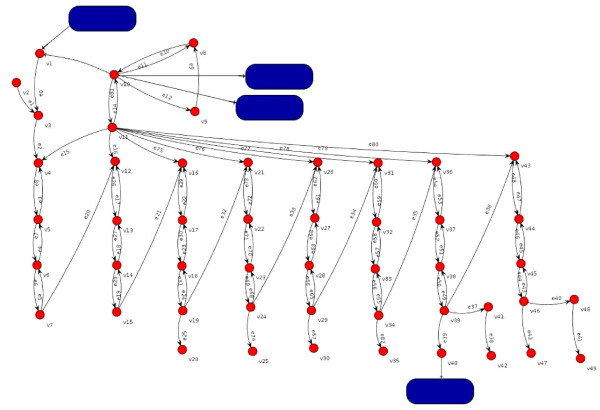
**Fatty acid Biosynthesis modeled using Graph**. (Vertex v_3 _corresponds to Acetyl-[acp], Vertex v_10 _corresponds to Malonyl-CoA and Vertex v_11 _corresponds to Malonyl-[acp]). Blue boxes correspond to other pathways.

The mycolic acid network [9] was modeled using a digraph (Figure 1) in which vertices represent the metabolites and reactions/interactions between any two metabolites are represented by arcs (Figure 2). Let D = (V, A) be the digraph with vertex set V = {v_1_, v_2_, v_3_, ..., v_49_} and there are four other metabolic cycles connected to the vertices v_1_, v_10 _and v_40 _of the digraph D and arc set A = {e_1_, e_2_, e_3_,...,e_80_}. By selecting the vertices with maximum out-degree (i.e. number of arcs radiating from the vertex) first, a set S = {v_11_, v_10_, v_39_, v_15_, v_19_, v_24_, v_29_, v_34_, v_46_} can be generated in D. Deleting this set S from the digraph D refers to deleting all the vertices of S from D and arcs that have one end vertex in S. Let D* = (V*, A*) be the resultant digraph obtained, where V* = V-S and A* is the set of all arcs remaining in A, which has more than four components in D*, a disconnected digraph.

### Determination of nearness index

The nearness index for a vertex (protein) is the sum of all the inverses of the minimum path lengths to every other vertex in the graph [2]. For a particular vertex **v**_**i**_, the eccentricity of v_i _is the length of the path from the farthest vertex in the graph and would contribute least to the nearness index of **v**_**i **_because the inverse of the path length is added equivalently; vertices with more eccentricity have a lower nearness index. If the degree of vertex **v**_**i **_(**i**^th ^vertex of given graph **G**) is denoted by **d**_**i**_, then the nearness index of **v**_**i **_is given by:

The data were parsed using a java program that uses the list of minimum path lengths (obtained from visANT) as the input.

## Results

Data for assessing the pathway are available in the KEGG (Kyoto Encyclopedia of Genes and Genomes) pathway database and were used to obtain a flowchart of the fatty acid biosynthesis pathway of *Mycobacterium tuberculosis *H37Rv. The green squares in the flowchart (Figure 1) represent proteins identified from the genome sequence of the bacterium. The genome sequence for this bacterium is complete. Therefore, the information concerning the participating proteins is also complete [10].

**Figure 3:**
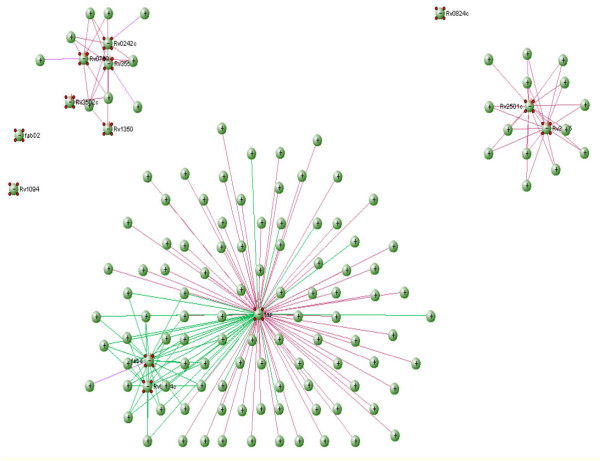
**Protein interaction network. Nodes have been colour coded and proteins which are closer are clustered together**.

The proteins involved in the mycolic acid biosynthesis pathway [9] were inputted into visANT software [11]. This free software can be used to identify interactions between proteins and the result is displayed as a graph. visANT can be downloaded from http://visant.bu.edu/. Programming for assessing the results was carried out using java (version 1.6.0_01). NetBeans IDE 6.9.1 was the IDE (Integrated Development Environment) used [12]. The name of the organism was selected and the list of proteins entered in the input area provided. Clicking the search button provides the interactions among the proteins given as the input. A graph is obtained and when the layout "spring embedded" is selected, the following is produced: Figure 3.

**Figure 4:**
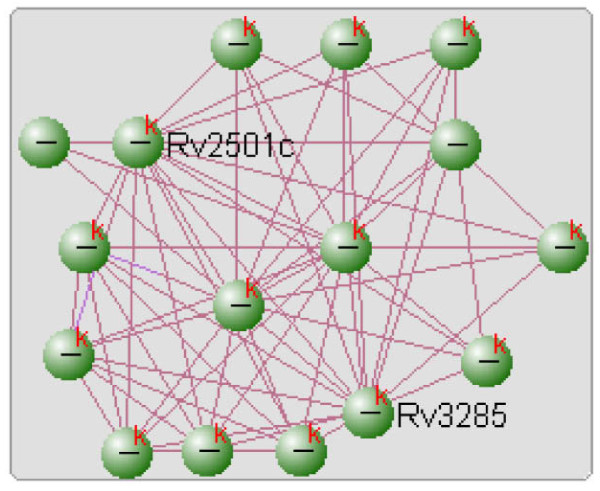
**Protein network demonstrating dense inter-connectivity**. visANT possesses a tool to identify the shortest paths between all the pairs of vertices in a graph. Selecting all the vertices of this small graph identifies the shortest paths. The shortest paths are given in the following format:

visANT represents proteins as vertices and the interactions between them as edges. Therefore, an undirected graph of the interactions of proteins involved in the fatty acid biosynthesis pathway is obtained. The graph is not directed as demonstrated in Figure 4.

**Figure 5:**
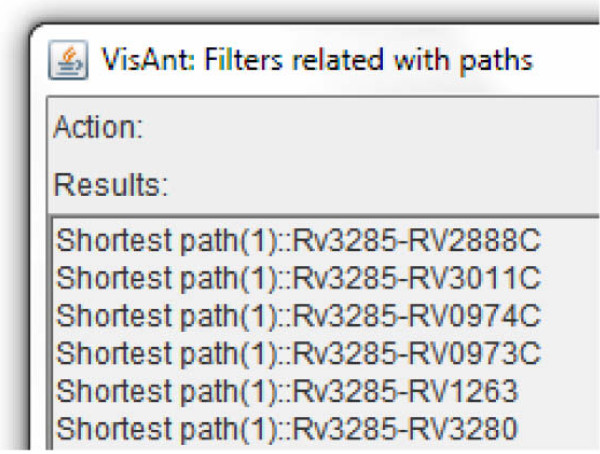
**Shortest Pathway analysis.** The shortest paths between all the pairs of vertices in a graph (Source: visANT).

This result as shown in Figure 5. can be stored in a text file and parsed to obtain the path distance (starting protein and the ending protein). For example, for the line Shortest path (2)::RV1384-RV2967C-RV0263C, the shortest path from protein RV1384 to protein RV0263C, is of length 2. This datum can be used to obtain the degree of each vertex. Therefore, when all the degrees have been determined, the amount of influence a protein possesses, using the concept of nearness index, can be calculated.

### Nearness index

The nearest vertex (with small ecentricity) would contribute most to the nearness index. Therefore, when calculating the nearness index, all the vertices of the graph were taken into account as were their differing levels of influence. The total sum represents the influence of the protein represented by **v**_**i **_on the complete pathway, which is represented as a graph. Another interaction between proteins concerns one protein helping in the production of a metabolite, which is converted to another metabolite by a second protein (2). In this case, the first protein influences the second protein. The second protein relies on the first protein functioning so that it obtains the metabolites. The graph obtained from protein-protein influences of this kind will be a directed graph. Reversible reactions are represented as edges to and from the two proteins. From this, the influence of each protein can be estimated by calculating its nearness index (Table 1). The result of shortest paths obtained from visANT can be copied and stored in a file called *shortpath*, which is given as an argument for running the java program. A java code like that shown below was written to calculate the nearness index with the file *shortpath *as the input.

**Table 1:** Protein nearness index

Protein	*N*_*i *_**(Nearness Index)**
**RV2501C**	15

**RV2967C**	15

**RV0973C**	15

**Rv3285**	14.5

**RV0263C**	12

**RV3280**	11.5

**RV2502C**	11.5

**RV3799C**	11.5

**RV0974C**	11.5

**RV2247**	11.5

**RV2888C**	10

**RV3375**	10

**RV3011C**	10

**RV1263**	10

**RV2363**	10

**RV1384**	9

   package nearnessindex;

   **import **java.util.Scanner;

   **import **java.util.regex.*;

   **import **java.io.*;

   /**

   *

   * **@author **Veeky

   */

   public class Main **{**

      static double i,iT**=**0;

      static String p1**=**"",p2**=**"", pT**=**"", topper;

      public static void main**(**String**[] **args**) {**

         //calculate nearness Index!

         try{

            PrintStream out **= new **PrintStream**(new **FileOutputStream**(**"indices"**));**

            System.setOut**(**out**);**

            Scanner s **= new **Scanner**(new **File**(**args**[**0**]));**

            **while (**s.hasNextLine**()) {**

            s.findInLine**(**"Shortest path\\((\\d+)\\)::(\\w+).*-(\\w+)"**);**//at each line, look for this pattern

            MatchResult result **= **s.match**();**//results from

            **if ((**p1.equals**(**result.group**(**2**)))&&(!**p2.equals**(**result.group**(**3**)))) {**

               i **= **i **+ (**1**/**Double.parseDouble**(**result.group**(**1**)));**

               p2 **= **result.group**(**3**);**

               }

            **else if (!**p1.equals**(**result.group**(**2**))) {**

               **if (!**p1.equals**(**""**)) {**

                  out.println**(**p1 **+ **": " **+ **i**);**

                  **if (**iT**<**i**) {**

                     topper **= **p1;//topper is the string that would hold the proteins with highest nearness index

                     iT **= **i;

               }

                  **else if (**iT**==**i**) {**

                     topper **= **topper **+ **", " **+ **p1;

               }

            }

            i **= (**1**/**Double.parseDouble**(**result.group**(**1**)));**

            p1 **= **result.group**(**2**);**

            p2 **= **result.group**(**3**);**

      }

      s.nextLine**();**

   }

   out.println**(**p1 **+ **": " **+ **i**);**

   out.println**(**"Protein(s) with highest nearness index (" **+ **iT **+ **"): " **+ **topper**);**

   s.close**();**

   out.close**();**

   }

   **catch (**FileNotFoundException e**) {**

      System.out.print**(**"cannot find file"**);**

      }

   }

}

### Computation of nearness index with Java code

In the main method of the program, a new *PrintStream *object was used to print to an output file called *indices*. A *scanner *object was used to read from the input file, *shortpath*, line by line. A while loop was used to check if *shortpath *was read up to the last line.

Inside the while loop, for each line a regex function (Shortest path\\((\\d+)\\)::(\\w+).*-(\\w+)) was used to identify the starting protein of the path, the ending protein and the path length. For instance, for the line Shortest path(2)::RV1384-RV2967C-RV0263C, we would obtain the result group(1) as 2, result group(2) as RV1384 and result group(3) as RV0263C. The shortest paths from each protein were calculated and according to the formula for the nearness index, the inverses of the shortest path length could be added. Redundant data concerning the various paths from one protein to another were discarded:

Shortest path(2)::RV2380C-RV2246-RV0099

Shortest path(2)::RV2380C-RV2245-RV0099

Shortest path(2)::RV2380C-RV1454C-RV0099

Shortest path(2)::RV2380C-RV0149-RV0099

Shortest path(2)::RV2380C-RV2381C-RV0099

Therefore, only one of these paths was considered as they were all considered to be the shortest.

Using visANT, the shortest path from each protein in to all the other proteins (of the form Shortest path(2)::RV1384-RV2967C-RV0263C) were obtained. This shortest path was used as an input for the java program to calculate the nearness index presented in Table 1.

**Figure 6:**
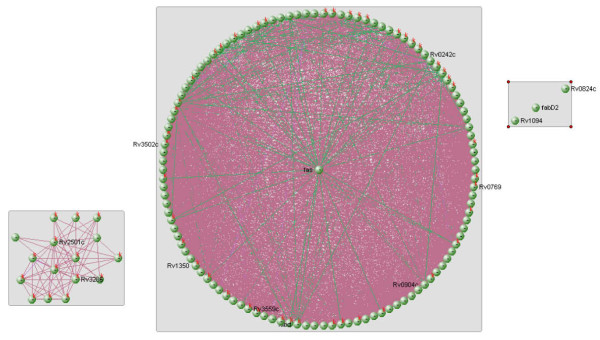
**Protein networks and their interactions (Source: visANT)**.

The proteins in Figure 6 with the highest nearness index of 15 were RV2501C, RV2967C and RV0973C. According to computational factors (namely domain fusion, gene neighborhood and phylogenetic profiling), these were the most influential proteins in this particular sub-graph. Among these proteins, RV2501C is directly involved in the fatty acid biosynthesis.

**Figure 7:**
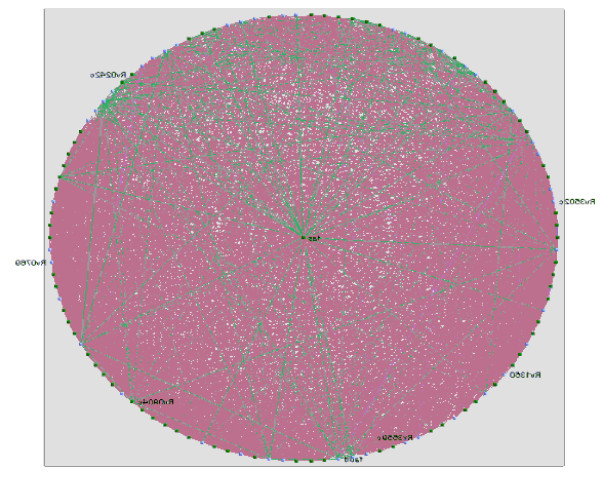
**The largest sub-graph**. This graph was used to calculate the nearness index of proteins.

For the largest sub-graph (Figure 7), the proteins with the largest nearness index of 104.4167 were RV2524C (FAS), RV2245 and RV2246. Of these proteins, FAS is directly involved in the pathway as shown in Table 2.

**Table 2:** Nearness Index For the largest sub-graph

Protein	***N***_***i***_**(Nearness** Index)	Protein	***N***_***i ***_**(Nearness** Index)	Protein	***N***_***i ***_**(Nearness** Index)
**RV2524C**	104.4167	**RV3826**	75.25	**RV0533C**	60.85

**RV2245**	101.4167	**RV1925**	74.75	**RV1108C**	60.35

**RV2246**	100.9167	**RV2941**	74.75	**RV3246C**	59.35

**RV2048C**	91.25	**RV0166**	74.75	**RV2884**	59.35

**RV2935**	91.25	**RV3801C**	74.75	**RV3765C**	59.35

**RV2934**	91.25	**RV1427C**	74.75	**RV0903C**	59.35

**RV2933**	91.25	**RV1058**	74.75	**RV0491**	59.35

**RV2932**	91.25	**RV3515C**	74.75	**RV1027C**	59.35

**RV2931**	91.25	**RV1206**	74.75	**RV0981**	59.35

**RV3825C**	91.25	**RV2383C**	74.68333	**RV0757**	59.35

**RV2940C**	91.25	**RV3561**	72.25	**RV0602C**	59.35

**RV3800C**	91.25	**RV1185C**	71.75	**RV1033C**	59.35

**RV1181**	91.25	**RV2948C**	71.75	**RV0818**	59.35

**RV0405**	91.25	**RV2930**	71.75	**RV2783C**	58.68333

**RV1527C**	91.25	**RV0035**	71.75	**RV2727C**	58.68333

**RV1664**	91.25	**RV2950C**	71.75	**RV3907C**	58.18333

**RV1662**	91.25	**RV1193**	71.75	**RV3105C**	58.18333

**RV1661**	91.25	**RV0404**	71.75	**RV1630**	58.18333

**RV2381C**	90.75	**RV1013**	71.75	**RV2539C**	56.85

**RV2048C_12**	90.75	**RV3667**	71.68333	**RV3607C**	56.35

**RV2946C**	90.75	**RV2590**	71.68333	**RV1409**	56.35

**RV2382C**	89.08333	**RV2505C**	71.25	**RV2244**	54.18333

**RV2947C**	89.08333	**RV3506**	71.25	**RV2214C**	54.08333

**RV1180**	89.08333	**RV1750C**	71.25	**RV3389C**	53.08333

**RV1483**	87.33333	**RV0542C**	71.25	**RV3538**	53.08333

**RV1663**	86.18333	**RV0551C**	71.25	**RV1938**	51.75

**RV3141**	84.68333	**RV1345**	71.25	**RV3617**	51.75

**RV3777**	84.68333	**RV2384**	70.18333	**RV0134**	51.75

**RV0149**	84.68333	**RV2380C**	69.68333	**ACCD3**	47.33333

**RV1454C**	84.68333	**RV0101**	69.68333	**RV0769**	39.78333

**RV1912C**	84.68333	**RV3513C**	68.75	**RV3559C**	39.78333

**RV2048C_11**	80.18333	**RV2187**	68.68333	**RV0242C**	39.78333

**RV0099**	77.25	**RV1550**	68.68333	**RV3502C**	36.61667

**RV0214**	77.25	**RV2379C**	66.68333	**RV1350**	36.61667

**RV1529**	76.75	**fabd**	64.85	**RV0768**	28.93333

**RV3089**	76.25	**RV2457C**	63.85	**RV0241C**	28.93333

**RV0270**	76.25	**RV3606C**	63.35	**RV3560C**	28.93333

**RV0119**	76.25	**RV1629**	63.35		

**RV1521**	76.25	**RV1018C**	60.85		

## Discussion

One of the pathways of *Mycobacterium tuberculosis *concerns fatty acid biosynthesis, and contributes to the synthesis of mycolic acid. The outer lipid layer (cell wall) of the bacterium makes it difficult for broad spectrum antibiotics to have any effect [4], and a major component of the cell wall is mycolic acid. Therefore, when synthesis of mycolic acid is reduced, broad spectrum antibiotics would be more effective owing to cell wall damage. Mycolic acid synthesis is the target of well-known anti-tuberculosis drugs including isoniazid, ethionamide and thiocarlide [9]. This suggests that any reactions that contribute to synthesis and processing of mycolic acids are viable targets for new drug discovery. visANT represents proteins as vertices and the interactions between them as edges, and there are various interactions between proteins involved in the fatty acid biosynthesis pathway.

Ranking proteins by the topological properties of the human protein-protein interaction net-work is one strategy for drug-target identification [13]. Another approach characterizes the interaction properties in protein-protein complexes; for example, identifying the domains involved in binding or analyzing the 3D structure. Comparison of domain-domain interactions and interfaces across an interactome can identify selective drug targets or drugs targeting multiple proteins (to block parallel pathways in a network) [14]. Structural analysis can be carried out to identify pockets where drugs could bind and to compare their properties with binding pockets on other proteins in the network [15].

The shortest and alternate paths in the reaction networks were examined. In an earlier study, sub-cluster profiling demonstrated that reactions in the mycolic acid pathway of mycobacteria form a tightly connected sub-cluster. Identification of hubs revealed that reactions involving glutamate were central to mycobacterial metabolism, and those involving pyruvate were at the centre of the *E. coli *metabolome. The analysis of shortest paths between reactions has revealed several paths that are shorter than well-established pathways. Using a directed graph to represent pathways would enable researchers to determine the importance of various proteins in a pathway and how their removal would affect that pathway [11].

The graph nodes represent metabolites and the edges represent enzymes. Based on an extended form of the graph theory model of metabolic networks, metabolite structural information was used to calculate the k-shortest paths between metabolites (the presence of more than one competing path between substrate and product). On the basis of these paths and connectivity information, load points were calculated and used empirically to rank the importance of metabolites/enzymes in the metabolic network. The load point analysis emphasized the role that the biochemical structure of a metabolite, rather than its connectivity (hubs), plays in the conversion pathway.

### Conclusions and scope

The graph of the fatty acid biosynthesis of *Mycobacterium tuberculosis H37Rv *had two major sub-graphs. The largest sub-graph had greater influence and its proteins would be better drug targets than the smaller sub-graph. When analyzed, this large sub-graph demonstrated that the protein that most influences the pathway is *FAS (Fatty Acid Synthase)*.

As demonstrated in Figure 2, the nearness indices of the proteins involved in the digraph can be determined. This process can be carried out on the directed graph obtained from protein dependences based on metabolites. For this, using visANT would require a line of input for each edge and this would be a cumbersome task. Therefore, alternative software such as Cytoscape is utilized. The KEGGraph library of R language provided by Bioconductor can be used to assess KEGG pathways in the form of graphs. This library takes KGML data for a given pathway and parses it to provide graphs. Using the resources available through visANT, the data of any biological pathway that is present in the KEGG database can be analyzed, and using a simple java program the most influential proteins in the pathway can be identified *in silico.*

## Competing interests

The authors declare that they have no competing interests.

## Authors' contributions

VB contributed in pathway modelling, programming and applying graphs in biology. UR analysed the biological data. TK was involved in applying graph theoretic approach. All authors read and approved the final manuscript.
